# Psychosocial Issues in Palliative Care: A Review of Five Cases

**DOI:** 10.4103/0973-1075.73642

**Published:** 2010

**Authors:** Tonia C Onyeka

**Affiliations:** Department of Anesthesia, Pain and Palliative Care Unit, University of Nigeria Teaching Hospital, Ituku-Ozalla, Enugu, PMB 01129, Nigeria

**Keywords:** Breaking bad news, Denial, Depression, Palliative care, Psychosocial issues, Stigma

## Abstract

Palliative care is not just vital in controlling symptoms of the patient’s disease condition, but also aims to extend the patient’s life, giving it a better quality. However, several times in the course of management, the psychosocial impact of cancer, HIV/AIDS, and other life-limiting disease conditions may not be noticed and dealt with during the admission period, thereby giving rise to a more complex situation than the disease condition itself. This article aims to review some psychosocial issues and measures that can be taken to address them. It highlights the various roles and the importance of the clinician, nurse, social worker, and other members of the multidisciplinary team in tackling these issues and will help healthcare professionals in this field achieve better practice in the future.

## INTRODUCTION

Care of the dying patient has become a specialized discipline within the medical field. Psychosocial care, as defined by the National Council for Hospice and Specialist Palliative Care Services, is care concerned with the psychological and emotional well-being of the patient and their family/carers, including issues of self-esteem, insight into an adaptation to the illness and its consequences, communication, social functioning and relationships.[1] It is a form of care that encourages patients to express their feelings about the disease while at the same time providing ways by which the psychological and emotional well-being of such patients and their caregivers are improved.[1] It has been suggested that because there may be a conflict among healthcare professionals (physicians, nurses, social workers, psychological counselors) on whose role it is to assist the patient with psychological, emotional, spiritual, and social concerns,[2] a large number of the patients’ needs remain unmet. In this article, six issues are reviewed in relation to five individuals admitted into the two-year old Pain and Palliative Care Unit of the University of Nigeria Teaching Hospital, Ituku-Ozalla, Enugu, Nigeria.

## STIGMATIZATION

For K.D., his first presentation to our clinic was at the age of 40 years, with multiple large grossly disfiguring facial, trunk, and limb tumors, where a diagnosis of neurofibromatosis type 1 was made. His cousin accompanied him from their village, about a three-hour drive, to the clinic on that visit but subsequently he defaulted from treatment. Several months later when the unit succeeded in tracking him down, it was discovered that each time he approached commercial drivers at the station where he was to get a ride to Enugu, the drivers declined. Even when he offered to pay for all the seats, so as to be driven alone, the matter was not any better. To make matters worse, passengers shunned him and passers-by stared at him, making jeering remarks while children taunted him.

Stigma can be said to be society’s negative evaluation of the particular features or behavior of certain individuals.[3] It exists not only for the cancer patient but also in persons with obvious deformities. When a person is stigmatized, his/her physical flaws become a negative form of identity by which several inappropriate reactions are elicited from the society. These reactions usually emanate from close associates, neighbors, and even strangers and may be echoed by the media. Neurofibromatosis type 1 has far-reaching physical, emotional, and social stigma associated with it. Because of the severe disfigurement, much attention is drawn to the patient, turning him/her into a spectacle and increasing psychological stress thus giving rise to social morbidity. Joseph Merrick, the famed “Elephant man” suffered abandonment and rejection by family and received the nickname for his gross physical disfigurement from neurofibromatosis type 1.[4] In many cultures where esthetic beauty is much sought after in order for an individual to obtain lucrative employment opportunities, such conditions become severe limitations and stumbling blocks. In time past, stigmatization even had a place in the law of some nations, forbidding persons with physical deformities to be seen in public.[3] Like this patient, individuals with disfigurement as a result of illness tend to feel isolated and are usually depressed. This affects their self-worth which in turn affects their interpersonal relationships, giving rise to withdrawal from family and community. Sometimes, in trying to seek a solution to their situation, they consult alternative medicine practitioners who have nothing to offer but rather increase their frustrations and despair.

How can patients successfully cope with stigmatization of their condition? Firstly, at the point a diagnosis is made, stigma should be identified and psychological counseling instituted along with treatments for the primary condition.[5] The social worker should organize family support groups, to get the patient and his family in contact with similar patients and their families. Such a forum would encourage an increase in medical knowledge on the disease condition, allow the exchange of experiences, as well as promote good interpersonal and family relationships. In addition, social worker and other members of the multidisciplinary team should encouraging open communication among family members as this will allow the patient express his fears and concerns about his condition. Advocacy is important as it will bring awareness of the condition to the society. This can be achieved through the dissemination of medical information via print and electronic media and the organization of workshops and seminars.

## DENIAL OF THE CANCER DIAGNOSIS

R.M. was a 25-year old final year undergraduate of veterinary medicine, referred to the unit by otolaryngologists with advanced nasoantral carcinoma. She had received several courses of cytotoxic treatments and together with her mother had received counseling from our unit. However, her mother refused to accept the diagnosis and insisted it was wrong, believing her daughter would be cured eventually.

It is not unusual for cancer patients or their families to reject a diagnosis or hope that it was a misdiagnosis. The phenomenon of denial in cancer patients is simply a mechanism by the patient, family members or both to avoid the reality of the illness. In many cases, this may lead to issues like refusal of patient to talk openly about the disease, avoidance of terminology associated with cancer during speech, diagnostic delay, treatment non-compliance,[6] refusal of further medical intervention or even defaulting of treatments. The term “denial” when defined from a psychoanalytic viewpoint can be an unconscious ineffective defense mechanism against painful and overwhelming aspects of reality.[7] When defined from the cognitive, stress, and coping model, it is regarded as an adaptive strategy (which may be conscious or unconscious) to protect the individual against painful events, perceptions, information, and feeling.[7,8]

There are two major forms of denial expressed in literature - adaptive and maladaptive denials.[9] They include the denial of diagnosis, the denial of impact, the denial of affect, psychotic denial and behavioral escape, amongst others.[7,9] In one study, denial of diagnosis was reported in a significant number of cancer patients.[10] Research on denial show that it produces varied outcomes on the patient. It can be a positive influence on the quality of life in some instances or may be negative in others.[7] It can cause patients to report fewer physical complaints that are not commensurate with the stage of the cancer or the treatment.[11] Other times, it may cause individuals to believe a miracle would occur, that the diagnosis would be wrong, and to attempt to engage in activities that had previously been discontinued as a result of the disease. For example, the patient with lung cancer may continue with the smoking habit. With further progression of the cancer, some patients wallow further in depression, despair, anxiety, anger, nervousness, and irritability, while others may continue in denial, even in the face of impending death.[7] Such patients present challenges to their medical teams and families and sometimes may require psychiatric evaluation even in the absence of identifiable psychiatric disorder.[8]

In managing denial, it is important for clinicians to eliminate lack of information or lack of understanding of the disease condition as a contributory factor.[12,13] Healthcare workers and caregivers should understand that patients have individualized requirements for receiving and processing information and various ways of coping with diagnosis.[14] Factors such as age, gender and educational level, cultural and social values may play a role. When patients receive adequate information, they are better equipped to accept the diagnosis and face the prognosis. Next, determine the type of denial (adaptive versus maladaptive), its usage, benefits, and risks to the patient. Avoid outright condemnation of the behavior. For instance, denying a cancer diagnosis may result in a refusal or delay in intervention (maladaptive), while a denial of impact (e.g. “I can carry out all activities as before the diagnosis”) may help maintain patient’s morale, cause him to comply with treatment instructions and go on with his life (adaptive). Intervention should be sought when the denial adversely affects the patient’s well-being. In the face of denial, clinicians should show empathy and maintain the relationship by seeing the patient often. The latter feel abandonment easily and therefore need to know that they are not in it alone. However, clinicians should avoid being too personally involved so as not to lose perspective on the seriousness of the illness or treatment options as a result of the development of emotional self-protection or “clinician denial”.[9] Family support interventions involving the social worker may be necessary while pharmacological treatment using anxiolytics and antidepressants can provide symptomatic relief.[9] Where there is a possibility of self-harm, harm to others or worsening of symptoms despite the treatments given, a psychiatric consult is crucial.[9]

## BREAKING BAD NEWS AND DEPRESSION IN THE CANCER PATIENT

O.C. was a 60-year-old female who was brought from the rural area to our hospital by her youngest daughter. Following assessment by the gynecologists, she was booked for Examination Under Anesthesia (EUA) and excision biopsy for suspected carcinoma of the cervix. On waking up in the recovery room, she discovered the mass was still present. When she enquired, she was told by a doctor on the team that there was nothing that could be done for her. From that moment, she became depressed and withdrawn, refusing to speak to anyone, including her children. That same day, she began to reject her meals, medications, and became excessively sleepy. When our unit visited subsequently, her condition remained unchanged. One afternoon, four days later, her daughter discovered she had passed on in her sleep.

Informing a patient of the diagnosis of his condition has become an art. Previously, many clinicians had no undergraduate or postgraduate training in breaking bad news. Prior to this present era of advanced cancer treatments, it had been the norm to both omit necessary information and give patients and relatives false hope while hiding the facts or explain to family members while keeping the patient in the dark. Only the rare brave few dared to “tell all” but in doing so, caused a lot of anger, depression, and distress for such patients and their families by their poor delivery techniques. Many patients end up severely and irreversibly traumatized by the attempt. The hippocratic oath is the earliest record of man’s attempt at defining a code of moral conduct for physicians, emphasizing the importance of upholding ethical standards of medical practice. But breaking the news of a cancer diagnosis can be devastating to the patient with far-reaching consequences.

Bad news has been defined as any information that worsens an individual’s point of view about their future and which has the potential to cause significant mental and behavioral problems.[15] It may be life-threatening (as seen in malignancy cases) or result in a temporal interruption of activities. It has been likened to the “dropping a bomb”[16] and is particularly difficult in the hands of the inexperienced clinician or in cases with poor prognosis.[16] The manner in which bad news is conveyed to the patient can affect clinical outcomes, interpersonal and family relationships, level of hopefulness,[17] and psychological adjustment.[18] However, such communication must be made in order to uphold the principles of informed consent and patient autonomy. Also, studies conducted in Scotland have revealed that 91% of cancer patients wanted to know the chances of a cure, indicating an increased need by such patients to know the truth about their illness.[19]

Barriers to effective communication between the physician and his patient where breaking bad news is concerned include superstition, cultural beliefs, misconceptions, social problems, and ignorance.[20] Others are language barrier (emphasizing the importance of medical translators)[21] and the ‘MUM” effect where the clinician is stressed by the bad news, creating a reluctance to speak of it to the patient.[16] Baile and his colleagues have formulated a six-step protocol, called “SPIKES’, to guide physicians in the process of breaking bad news.[16] It involves: 1. Setting up the interview (privacy, comfort, and family involvement); 2. Assessing the patient’s perception of his condition; 3. Obtaining the patient’s invitation to divulge information. 4; Giving knowledge and information to the patient; 5. Addressing the patient’s emotions with empathic responses; 6. Strategy and summary (presenting treatment plan and options). It has been suggested that the information of the patient’s diagnosis and treatment options should be in the written form.[16]

Depression is an emotional state and a form of psychological distress that commonly occurs in patients with life-threatening illness. It is associated with significant mortality and morbidity[22] and can drastically alter any meaningful palliative care treatments rendered to the patient. It is estimated to have a prevalence rate of between 3 and 45%,[23] a situation existing because of the unavailability of an appropriate screening tool. But also, because many conclude that depression is a natural inevitable reaction to terminal illness, it usually goes undiagnosed and untreated in a good number of patients.[22] It is known to be a predictor of desire for death in the terminally ill patient.[23] Many barriers exist to the detection of depression in vulnerable patients. Somatic symptoms of depression such as pain can resemble the symptoms of the physical disease while many clinicians view depression as an inevitable part of the dying process.[24] Lack of proper communication skills such as patient-centered consulting and active listening are absent, while poor communication between palliative care physicians and psychiatrists can also be contributory.[24]

Causes of depression include knowledge of a life-threatening diagnosis, presence of physical symptoms like pain and nausea, side effects from medical treatments, and loss of independence and functionality. Others are changes in family relationships, concern for dependents and changes in bodily function.[25] Patients who are usually at risk of developing depression (apart from a family history of depression) include patients whose symptoms are poorly controlled or those who have poor communication with their healthcare provider, among others.[25] Symptoms of depression are categorized in two groups: somatic and psychological. These patients usually manifest symptoms such as fatigue, sleep disturbances (excessive sleep or insomnia), reduced appetite, being less talkative, and may be tearful. He characteristically has a withdrawn mood, loss of interest in his environment and in activities. He may have a desire or Wish To Hasten Death (WTHD)[26] or be suicidal in intent. There is presently a lack of consensus concerning the ideal screening tool for depression,[24] but common tools in use include the Brief Edinburgh Depression Scale and the Hospital Anxiety and Depression scale (HADS).

Depression is treatable. Psychological support may be adequate in mild cases while suitable pain relief might improve symptoms considerably.[27] For clinicians to avoid the creation of depression in patients with terminal illness, they should apply well-established principles of communication and counseling when breaking bad news,[16] give the individual the information in the manner he desires and allow for the open expression of emotions. Involvement of specialized palliative care nurses and social workers to give supportive psychotherapy[28] as well as the involvement of family members and religious leaders can help in the care process. Where the depression persists for several weeks despite non-drug interventions or where a definite depressive syndrome has been identified, assessment by a psychiatrist and drug treatments are indicated. Tricyclic antidepressants (e.g. lofepramine) are useful with their added ability to cause sedation and anxiolysis in the patient but they have anti-cholinergic side effects too. Selective serotonin reuptake inhibitors (e.g. sertraline) have fewer anti-cholinergic side effects but cause diarrhea and headache.

## UNRESOLVED FAMILY CONFLICTS

Fifty-one-year-old C.A., whose carer was his younger sister, was severely depressed as he had been abandoned for several weeks by members of his immediate family. When nurses in our palliative care unit interviewed him, he gave the reason for the abandonment as being his change from the family religion to a different one of which his wife and three adult sons never approved of. But on investigation, it was discovered that his wife and sons were bitter about his abandonment of the family in their earlier years and subsequent involvement in several extramarital affairs, deciding that he did not deserve any care or help from them.

The cancer diagnosis is a situation that affects not just the patient but also family members and other caregivers, producing great degrees of psychosocial distress. All parties involved have several unmet needs. The interplay of the relationships involved produces a lot of moral obligations and responsibilities. In addition, there are emotional and physical stresses affecting the patient and his family members/caregiver. This is more pronounced in many developing nations where palliative care is still at the stages of infancy, and the full complement of multidisciplinary team and support services are grossly inadequate or lacking.

Interpersonal and family relationships can affect chronic disease management outcomes.[29] The psychological mechanisms involved in the disease process can be influenced by either the calming effects of secure and harmonious family attachments or the disruptive effects of family enmity and criticism.[29] For instance, the quality of care for the patient is adversely affected by divorce or separation.[30] Several articles have enumerated the many patient and caregiver needs such as loss of autonomy, financial and future problems, spiritual and informational needs, among others. Some have highlighted the existence of family discord with regards to care and decisions at the end-of-life care. But there is a paucity of research concerning the role of old family conflicts, unsettled disputes and disagreements (i.e. those existing before the illness) as they affect the quality of patient care and the disease process. More studies are encouraged in this area.

A family-focused intervention directed at crisis intervention and promotion of family unity is recommended to help improve family relationships. One commonly used approach is a group-based educational and behaviorally focused problem management technique that will help foster emotional expressiveness, prevent disease domination of the family life and promote conflict resolution.[29] Another is family or couple psychotherapy, where social workers intervene together with the use of family education support groups.[31] Family members must be carried along in the informative and decision-making process in the course of treatment. Members of the multidisciplinary team should be encouraged to take courses on conflict resolution and on advanced communication skills in palliative care.

## CANCER IN THE PHYSICALLY CHALLENGED

N.O. was a 19-year-old girl diagnosed with advanced oropharyngeal malignancy. She was born deaf–mute and had been living with her parents, attending the local school for the hearing-impaired. Because there was no one trained in sign language, communication between the patient, the palliative care team and the otolaryngologists was extremely tasking. This sometimes caused the young girl to breakdown in tears, frustrated by her inability to be understood. During one of the several discussions between the palliative care nurses and her mother, it was discovered that the patient’s parents thought of the educational training of their deaf daughter as a waste of resources, and had been encouraged by their relatives to stop her studies. This attitude was a contributory factor to the late presentation of the patient to the surgeons for management of her malignancy.

Very little attention has being paid by palliative care to the needs of disabled people who are nearing the end-of-life.[32] A disability is anything that causes an inability of an individual to function normally, physically or mentally.[33] Hearing impairment is classified as an invisible disability because there may be no visual clues that the patient has any such impairment.[34] The cancer diagnosis alone is an emotionally overwhelming situation on its own. The vision or hearing-impaired cancer patient needs a sign language interpreter to help out in communication. Where such is lacking, he is not able to either hear his caregiver or respond adequately to questions. Many of such patients are forced to endure prejudice, bias, stigma, and marginalization, in addition to the disease condition they are suffering from.

Deafness is defined as a loss of hearing sufficiently severe to render an understanding of conversational speech impossible in most situations, with or without a hearing aid.[35] It is in many cases associated with speech impairment, especially if of the profound type. Hearing loss affects a wide range of situations in the patient’s life. Communication with friends and family is affected. Affected individuals often experience sub-optimal doctor–patient relationships as a result of this. They are prone to being withdrawn from social activities, and this in turn leads to reduced intellectual and cultural stimulation and eventually isolation and depression.[35] It has been said that hearing-impaired patients may pose a greater challenge in medical management of their conditions than the visually impaired.[34]

Encouraging the patient to identify with others who have similar disability and to experience companionship with others who have similar psychosocial needs will help him accept deafness as part of their identity and thereby help them accept themselves and acquire some self worth.[36] Healthcare providers should enhance communication with hearing-impaired patients that employ lip-reading by facing such patients directly and maintaining eye contact, keeping hands and other objects away from the mouth.[34]

Sign language interpreters should be employed to help in the communication process, especially for cases where the patient feels self-conscious or embarrassed discussing his disease with family members or friends as interpreters. However, in the absence of a professional interpreter, family members may be used. Watching patient’s facial expression for discomfort and other emotions can be helpful too.

## CONCLUSION

To achieve good palliative care, good psychosocial care is imperative. Presently, care of the cancer patient is moving from the patient-centered approach to the ’whole-system approach’[29] that encompasses the patient’s interpersonal and family relationships as well as the best of medical and social care, in order to optimize the quality of life for such patients. Stigmatization can prevent affected patients from seeking appropriate medical care and therefore must be dealt with. Members of the multidisciplinary team must arm themselves with good communication skills. In addition, healthcare providers must be careful not to unconsciously portray a sense of hopelessness to their patients with regards to the disease diagnosis or prognosis. Compassion and empathy should be their watchword. They should also keep themselves from dismissing anxiety and depression as understandable, thereby denying many of essential treatment. Finally, all procedures in palliative care should aim at ensuring the patient lives a life as comfortable as possible until death.
